# Risk factors and diagnostic indicators for congenital syphilis: a Nationwide retrospective survey

**DOI:** 10.1017/S0950268825100629

**Published:** 2025-10-22

**Authors:** Hiroyuki Shimizu, Munehiro Furuichi, Noriko Takeuchi, Kenta Ito, Takanori Funaki, Yoshinori Ito, Masaaki Mori, Hiroyuki Moriuchi, Takuya Yamagishi, Masayoshi Shinjoh

**Affiliations:** 1 Department of Clinical Laboratory Medicine, Fujisawa City Hospital, Kanagawa, Japan; 2 Department of Pediatrics, Keio University School of Medicine, Tokyo, Japan; 3 Department of Infectious Diseases, Medical Mycology Research Center, Chiba University, Chiba, Japan; 4 Department of General Pediatrics, Aichi Children’s Health and Medical Center, Obu, Japan; 5 Center for Research Planning and Coordination, National Institute of Infectious Diseases, Tokyo, Japan; 6 Department of Pediatrics, Aichi Medical University, Nagakute, Japan; 7 Division of Rheumatology and Allergology, St Marianna University School of Medicine, Kawasaki, Japan; 8 Department of Lifelong Immunotherapy, Institute of Science Tokyo, Tokyo, Japan; 9 National Research Center for the Control and Prevention of Infectious Diseases, Nagasaki University, Nagasaki, Japan; 10 Antimicrobial Resistance Research Center, National Institute of Infectious Diseases, Tokyo, Japan

**Keywords:** congenital syphilis, risk factor, rapid plasma reagin, Total IgM, maternal syphilis

## Abstract

This nationwide retrospective study in Japan aimed to identify risk factors and diagnostic indicators for congenital syphilis (CS) and improve diagnostic accuracy. Data were collected from 230 pregnant women diagnosed with syphilis and their infants between 2015 and 2024. Of these, 49 infants were diagnosed with definite or highly probable CS, while 73 infants with excluded CS served as the control group. Multivariable logistic regression analysis revealed two significant risk factors for CS: maternal treatment not completed more than 4 weeks before delivery (odds ratio [OR]: 7.20; 95% confidence interval [CI]: 1.38–37.56; p = 0.02) and elevated total IgM levels in the infant (>20 mg/dL) (OR: 65.31; 95% CI: 4.53–941.39; p = 0.002). When using infant rapid plasma reagin (RPR) ≥1 as a diagnostic indicator, sensitivity was 93.8% (n = 48). In contrast, the infant-to-mother RPR ratio ≥1 showed a lower sensitivity of 34.3%, with fewer cases available for analysis (n = 35) due to limited maternal data. These findings indicate that delayed maternal treatment and high total IgM levels in the infant are significant risk factors, while the infant’s RPR titre serves as a useful diagnostic indicator for CS.

## Introduction

Congenital syphilis (CS), caused by *Treponema pallidum*, is transmitted from mother to foetus and can lead to severe adverse outcomes [1]. Globally, CS remains a significant public health concern, not limited to low-income countries, with the World Health Organization aiming to reduce mother-to-infant transmission to fewer than 50 cases per 100,000 live births [2, 3]. In Japan, syphilis cases have increased since the early 2010s [4], driven by heterosexual transmission, leading to a rise in CS cases [5–8]. The calculated incidence rate of CS per 100,000 live births rose from below 1.0 before 2013 to 5.09 in 2023 [8]. Despite robust screening programmes, CS has been increasing in recent years in Japan, emphasizing the need for ongoing vigilance.

Early screening and treatment of pregnant women can prevent CS [9, 10], but approximately 70% of affected infants are asymptomatic at birth, and passively transferred maternal antibodies complicate early diagnosis and treatment [11]. Therefore, guidelines in Japan [12] and other countries [1, 13, 14] recommend estimating the likelihood of CS based on maternal and infant non-treponemal antibody tests, clinical symptoms, and maternal syphilis treatment history [13, 14]. However, the complexity of current guidelines often leads to overtreatment in an effort to avoid missed cases. More accurate diagnostic tools are needed, requiring a better understanding of each indicator’s diagnostic contribution.

To address these issues, we conducted the first nationwide survey to assess the aetiology of CS and analyse risk factors in both mothers and infants, aiming to enhance clinical guidelines [12].

## Materials and methods

### Study protocol and data collection

This nationwide retrospective cohort study included pregnant women with syphilis from 2015 to 2024 and followed the outcomes of their pregnancies. Recruitment was conducted by inviting member physicians of the Japanese Society for Pediatric Infectious Diseases between April and August 2024. Participating physicians provided anonymized clinical data from their institutions; patients did not self-register. Participants included pregnant women with a positive syphilis serologic test, regardless of disease activity, and their infants with a potential risk of CS. Data were collected on maternal age, social history (e.g., engagement with commercial sex work in the past or currently), syphilis stage, time of diagnosis, treatment details, and serological test results. Infant data included sex, perinatal information, clinical symptoms, and serological test results. After excluding those who did not consent, maternal and infant risk factors were analysed to identify contributors to diagnosis.

### Tests for diagnosis of CS

Serological testing is the most common method for diagnosing syphilis and is divided into non-treponemal (non-specific) and treponemal (specific) tests. The rapid plasma reagin (RPR) test was used as a non-treponemal test, and either *T. pallidum* latex agglutination or *T. pallidum* hemagglutination as treponemal tests. The automated quantitative RPR assay is widely used as it eliminates variability between laboratory technicians, making it more objective and easier to monitor antibody titre trends [12, 15, 16]. Although some facilities in our study still used the conventional manual card test, previous studies in the US and Japan revealed a strong correlation between the two methods, allowing both results to be treated as comparable [16–18]. Furthermore, the optimal decrement rate in RPR titre by the automated test for a 4-fold decrement by manual card test was reported to be 76.54% [16]. Therefore, given its superior speed, lack of interpersonal variation in interpretation, and higher throughput compared to the manual test, the automated assay is recommended in Japan as an excellent alternative. A positive result is indicated by a value greater than 1.0 R.U.

To explore alternative serum biomarkers for diagnosing CS, in addition to the infant-to-mother RPR ratio, we utilized the results of absolute RPR, total IgM, and fluorescent treponemal antibody absorption (FTA-ABS) IgM fractions of infants, which had been performed as part of routine clinical practice; among these, the FTA-ABS IgM was not covered by the national health insurance in Japan. However, these tests were not uniformly performed in all cases, and their implementation depended on the policy of each facility and attending physician.

### Final diagnostic definition of CS

Definitive diagnosis of CS is often difficult due to the presence of transferred maternal antibodies. The definition of confirmed CS varies depending on the country and organization [3]. Infants were classified into four major categories (A–D) based on the likelihood of CS. Each category was further subdivided according to the diagnostic criteria applied (e.g., A1, A2; B1, B2; C1, C2), resulting in seven patterns in total (Table 1). CS cases were categorized as follows: definite diagnosis included A1 (direct detection of *T. pallidum* in lesions, body fluids, or nasal discharge) [14] and A2 (detection of *T. pallidum*-specific IgM antibodies) [19, 20]. In placental pathology, identification was achieved using *T. pallidum* immunohistochemistry, after observing findings such as villitis. Highly Probable Diagnosis was assigned to infants with a positive non-treponemal test for syphilis and any of the following: clinical manifestations consistent with CS, radiographic evidence of long bone abnormalities, or abnormal cerebrospinal fluid analysis. Abnormal cerebrospinal fluid findings were defined, considering neonatal reference ranges, as a cell count ≥20/mm^3^ and a protein level ≥ 119 mg/dL [21]. Excluded diagnoses encompassed C1 (untreated infants with negative RPR testing after 3 months of age) and C2 (infants with negative treponemal serology confirmed before the age of 18 months) [22, 23]. The category C2 also included children who were considered unlikely to have CS but were treated. Cases that did not fit into any of the aforementioned categories due to an unclear diagnosis were designated as D (Undiagnosed). Maternal treatment history influences the likelihood of CS but is not part of the diagnostic criterion and is therefore excluded.

**Table 1.** Final diagnostic definition of congenital syphilis

Diagnosis	Laboratory test results related to congenital syphilis	Category
Definite diagnosis	Identification of *Treponema pallidum* by	A1
	• Darkfield microscopy of lesions, body fluids, or neonatal nasal discharge, OR	
	• PCR or other equivalent direct molecular methods of lesions, body fluids, placenta, umbilical cord, or autopsy material, OR	
	• Immunohistochemistry, or special stains (e.g., silver staining) of specimens from lesions, placenta, umbilical cord, or autopsy material.	
	Detection of *T. pallidum*-specific IgM (FTA-abs, EIA)	A2
Highly probable diagnosis	An infant or child who has a reactive non-treponemal test for syphilis (VDRL, RPR, or equivalent serologic methods) AND any one of the following:	B1
	• Any evidence of congenital syphilis on physical examination	
	• Any evidence of congenital syphilis on radiographs of long bones	
	• A reactive CSF VDRL test	
	• In a non-traumatic lumbar puncture, an elevated CSF white blood cell count or protein (without other cause)	
	Persistent positive treponemal serology in a child older than 18 months of age.	B2
Excluded diagnosis	Negative RPR after 3 months without treatment.	C1
	Negative treponemal serology was confirmed before the age of 18 months.	C2
Undiagnosed		D

Abbreviations: CSF, cerebrospinal fluid; EIA, enzyme immunoassay; FTA-abs, fluorescent treponemal antibody-absorption; IgM, immunoglobulin M; PCR, polymerase chain reaction; RPR, rapid plasma reagin; VDRL, venereal disease research laboratory test.

In Japan, CS is also a notifiable disease under the national surveillance system, which has its own notification criteria (including: [i] markedly higher infant than maternal nontreponemal titres, [ii] persistently elevated infant titres beyond the expected decline of maternal antibodies, [iii] positive infant IgM against *T. pallidum*, [iv] clinical signs of early CS, and [v] signs of late CS) [24]. These criteria differ from the case definitions used in this study, which were specifically designed for research purposes.

### Potential predictors for CS

We evaluated potential predictors of CS by comparing definite or highly probable diagnoses with excluded diagnoses. Univariate analysis was conducted using Fisher’s exact test for categorical variables and univariate logistic regression analysis for continuous variables. A multivariate logistic regression analysis was then performed to identify potential predictors of CS. Considering multicollinearity and the appropriate number of variables for the multivariable analysis, the following covariates were included in the multivariate models based on their potential associations in the univariate analysis: *p-*values less than 0.05 were considered statistically significant. We also compared the results of serological tests (maternal and infant RPR, and infant RPR/maternal RPR) as diagnostic indicators between CS and non-CS cases.

### Statistical analysis

All statistical analyses were conducted using the SPSS Statistics 29.0 software (SPSS, Chicago, IL).

### Ethical considerations

This study was approved by the Keio University Ethics Committee (Approval Number 20231204, recently revised in 2024), with a waiver of informed consent because the data were anonymized. To ensure participant autonomy, an opt-out option was provided.

## Results

### Pregnant women with syphilis

**Figure 1.**
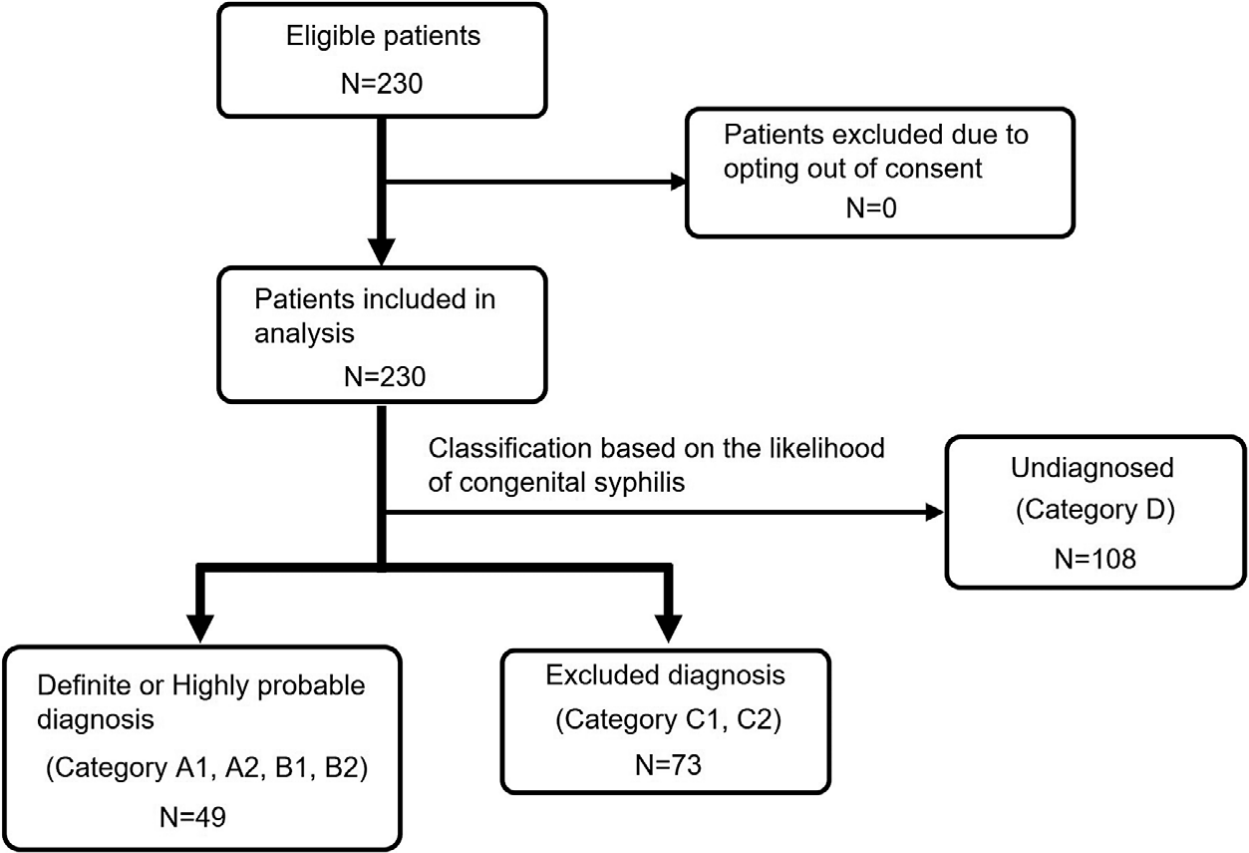
Flowchart of study participants. A total of 230 pregnant women with syphilis and their infants were included. No patients were excluded due to opting out of consent. Based on diagnostic criteria, 49 infants were classified as having a definite or highly probable diagnosis of congenital syphilis (Categories A1, A2, B1, B2), 73 were classified as excluded (Categories C1, C2), and 108 remained undiagnosed (Category D).

In all, 39 of the 42 (92.9%) facilities responded to the study’s questionnaire. A total of 230 pregnant women with syphilis during pregnancy were enrolled in this study (Figure 1). Table 2 presents maternal background, serological tests, syphilis stage, and treatment details.

**Table 2.** Clinical characteristics of pregnant women with syphilis

		*n* = 230	
Maternal background	Age at delivery	26.9	
	Unmarried at delivery	96/223	43.0%
	History of sexually transmitted infections^a^	60/173	34.7%
	Engaged as a commercial sex worker	55/173	31.8%
	Failure to receive prenatal health checkups or irregular visits	66/220	30.0%
	Syphilis stage		
	Latent syphilis (asymptomatic)	164/191	85.9%
	Latent syphilis (time of infection unknown)	119/191	62.3%
	Early latent syphilis (less than 1 year after infection)	20/191	10.5%
	Late-stage latent syphilis (more than 1 year after infection)	25/191	13.1%
	Primary syphilis (local symptoms such as initial induration and stiff chancre)	8/191	4.2%
	Secondary syphilis (systemic symptoms such as rash and lymphadenopathy)	19/191	9.9%
Maternal therapy	Treated with antimicrobials	197/222	88.7%
	Penicillin G (IV)	20/222	9.0%
	Penicillin G (IM)	35/222	15.8%
	Penicillin G (IV or IM)	55/222	24.8%
	Ampicillin/Ceftriaxone (IV)	9/222	4.1%
	Amoxicillin (PO)^b^	115/222	51.8%
	Amoxicillin + Probenecid (PO)	14/222	6.3%
	Maternal treatment completed more than 4 weeks before delivery	132/190	69.5%
Non-treponemal test results	Maternal RPR at delivery^c^	30.8	(2.9–18.45)
	Maternal RPR >4	123/206	59.7%

Abbreviations: IgM, immunoglobulin M; IM, intramuscular; IV, intravenous; PO, per Os; RPR, rapid plasma reagin.

a A documented history of sexually transmitted infections encompasses past diagnoses of conditions such as syphilis, trichomoniasis, chlamydial or gonorrheal infections, and genital herpes.

b In Japan, oral amoxicillin is frequently used for the treatment of syphilis, particularly because intramuscular benzathine penicillin G, the global standard treatment, was unavailable in the country until 2022.

c Median (interquartile range).

The mean maternal age was 26.9 years, with a median of 26 years. In total, 43% of mothers (96/223) were unmarried, and 34.7% (60/173) had a history of sexually transmitted infections other than syphilis. A total of 31.8% (55/173) were engaged as commercial sex workers, and 30.0% (66/220) had not received antenatal checkups or had irregular checkups. The stages of syphilis were as follows: asymptomatic latent syphilis 85.9% (164/191), primary syphilis 4.2% (8/191), and secondary syphilis 9.9% (19/191). Amoxicillin with or without probenecid was used in 58.1% of cases (129/222), while 24.8% (55/222) received penicillin G, including intramuscular formulations approved for use in 2022.

### Infants born to pregnant women with syphilis

A total of 230 infants were born to mothers with syphilis during the study period (Table 3). There were no cases of stillbirth or miscarriage. Among these infants, 127 were male and 101 were female; 170 were born via vaginal delivery and 58 via caesarean section. The average birth weight was 2,772 g, and 23.7% (54/228) of the infants were classified as low birth weight (< 2,500 g). Of these, 49 were diagnosed with definite or highly probable CS; 73 were determined not to have CS, and 108 had an unknown final diagnosis. Among the 49 definite or highly probable cases, 13 (26.5%) were diagnosed by placental histological identification of *T. pallidum*, 21 (42.9%) by FTA-ABS IgM, and 20 (40.8%) by a positive non-treponemal test result along with any of the following: physical abnormalities, long bone abnormalities, or abnormal cerebrospinal fluid findings.

**Table 3.** Clinical characteristics of infants born to mothers with syphilis

	*n* = 230	
Gender (male)	127/228	55.7%
Mode of delivery (cesarean section)	58/228	25.4%
Preterm	42/226	18.6%
Low birth weight	54/228	23.7%
Definite (A) or highly probable (B) diagnosis	49/230	21.3%
(A) Definite diagnosis	29/49	59.2%
(A1) Identification of *Treponema pallidum* by		
• Darkfield microscopy	0/49	0.0%
• PCR	1/49	2.0%
• Placental histopathology	13/49	26.5%
(A2) FTA-abs IgM	21/49	42.9%
(B) Highly probable diagnosis	20/49	40.8%
(B1) Positive non-treponemal test AND		
• Physical examination	29/49	59.2%
• Radiographs of long bones	13/49	26.5%
• CSF-positive VDRL	12/49	24.5%
• Abnormal CSF findings	33/49	67.3%
(B2) Persistent positive treponemal test in a child older than 18 months^a^	4/49	8.2%
Excluded diagnosis (C)	73/230	31.7%
(C1) Negative RPR after 3 months without treatment.	25/73	34.2%
(C2) Negative treponemal serology before 18 months.	56/73	76.7%

Abbreviations: CSF, cerebrospinal fluid; FTA-abs, fluorescent treponemal antibody-absorption; IgM, immunoglobulin M; PCR, polymerase chain reaction; RPR, rapid plasma reagin; VDRL, venereal disease research laboratory test.

a Including cases that meet higher-level definitions.

A total of 108 undiagnosed cases were excluded from the risk factor analysis, as most were ultimately lost to follow-up due to insufficient testing rather than insufficient surveillance. For example, 82.5% (33/40) had a negative infant RPR (<1.0 R.U.) at birth, and none of these cases underwent a repeat RPR test at 3 months. The antibiotic treatment rate was 56.1% (60/107), and the FTA-ABS IgM measurement rate, calculated as the number of patients measured divided by the total number of patients with known measurement status (i.e., whether measured or not), was 65.7% (36/105), and all were FTA-ABS IgM-negative. Furthermore, the total IgM measurement rate and infant RPR test rate were 62.0% (67/108) and 89.8% (97/108), respectively.

### Risk factor analysis for CS

To evaluate the potential indicators of CS, we compared cases with a definite or highly probable diagnosis to those with an excluded diagnosis (Table 4). In the univariate analysis, being engaged as a commercial sex worker (odds ratio [OR]: 5.33, 95% confidence interval [CI]: 2.12–13,40, *p* < 0.001), failure to receive prenatal health checkups or irregular visits (OR: 3.91, 95%CI: 1.76–8.68, *p* < 0.001), preterm birth (OR: 10.29, 95%CI: 3.90–21.14, *p* < 0.001), and low birth weight (OR: 13.78, 95%CI: 5.40–35.16, *p* < 0.001) were significantly associated with CS. The authors also found that certain blood test results (low platelet count, high CRP, high total IgM, and maternal RPR >4) were more commonly observed in the definite or highly probable CS group. Furthermore, infants born to mothers who had not completed treatment more than 4 weeks before delivery had a significantly higher rate of CS (OR: 16.92, 95%CI: 6.39–44.82, *p* < 0.001).

**Table 4.** A univariate analysis of predictors of congenital syphilis

		Congenital syphilis								
		Definite (A) or highly probable (B) diagnosis		Excluded diagnosis (C)						
		*n* = 49	%	n = 73	%	OR	95%CI			*p*
Maternal background	Age at delivery^a^	23.5	(22–27)	27	(24–31)	0.98	0.92	-	1.04	0.51
	Unmarried at delivery	29/48	60.4%	30/71	42.3%	2.09	0.99	-	4.40	0.06
	History of sexually transmitted infections	15/35	42.9%	26/59	44.1%	1.05	0.45	-	2.44	1.00
	Engaged as a commercial sex worker	20/35	57.1%	12/60	20.0%	5.33	2.12	-	13.40	<0.001
	Inadequate or no antenatal health checkups	25/47	53.2%	16/71	22.5%	3.91	1.76	-	8.68	<0.001
	Syphilis stage									
	Latent syphilis (asymptomatic)	32/38	84.2%	57/66	86.4%	0.84	0.28		2.58	0.78
	Latent syphilis (time of infection unknown)	20/38	52.6%	41/66	62.1%	0.68	0.30	-	1.52	0.41
	Early latent syphilis (less than 1 year after infection)	10/38	26.3%	8/66	12.1%	2.59	0.92	-	7.28	0.10
	Late-stage latent syphilis (more than 1 year after infection)	2/38	5.3%	8/66	12.1%	0.40	0.08	-	2.00	0.32
	Primary syphilis (local symptoms such as initial induration and stiff chancre)	1/38	2.6%	4/66	6.1%	0.42	0.05	-	3.89	0.65
	Secondary syphilis (systemic symptoms such as rash and lymphadenopathy)	5/38	13.2%	5/66	7.6%	1.85	0.50	-	6.85	0.49
Maternal therapy	Treated with antimicrobials	36/46	78.3%	68/73	93.2%	0.27	0.08	-	0.83	0.02
	Penicillin G (IV)	3/46	6.5%	13/71	18.3%	0.31	0.08	-	1.16	0.10
	Penicillin G (IM)	7/46	15.2%	9/71	12.7%	1.24	0.43	-	3.59	0.79
	Penicillin G (IV or IM)	10/46	21.7%	22/71	31.0%	0.62	0.26	-	1.47	0.30
	Ampicillin/Ceftriaxone (IV)	3/46	6.5%	4/71	5.6%	1.17	0.25	-	5.48	1.00
	Amoxicillin (PO)	18/46	39.1%	39/71	54.9%	0.53	0.25	-	1.12	0.13
	Amoxicillin + Probenecid (PO)	4/46	8.7%	4/71	5.6%	1.60	0.38	-	6.72	0.71
	Maternal treatment NOT completed more than 4 weeks before delivery	38/45	84.4%	17/70	24.3%	16.92	6.39	-	44.82	<0.001
Infant	Gender (male)	20/48	41.7%	33/73	45.2%	0.87	0.42	-	1.81	0.71
	Mode of delivery (cesarean section)	14/48	29.2%	17/73	23.3%	1.36	0.59	-	3.10	0.53
	Preterm	24/46	52.2%	7/73	9.6%	10.29	3.90	-	27.14	<0.001
	Low birth weight	31/49	63.3%	8/72	11.1%	13.78	5.40	-	35.16	<0.001
Test results of the infant	White blood cell count (/μL)^a^	16,195	(13160–20,370)	14,950	(12400–18,380)	1.00	1.00	-	1.00	0.38
	Hgb (g/dL)^a^	14.5	(10.75–16.8)	16.7	(15.8–18.3)	0.72	0.62	-	0.85	<0.001
	Platelet count (10×4/μL)^a^	11	(6.25–26.2)	28.85	(25.9–32)	0.90	0.86	-	0.94	<0.001
	T-bil (mg/dL)^a^	2.35	(1.75–2.95)	2.17	(2–2.8)	1.02	0.87	-	1.20	0.81
	AST (U/L)^a^	57.5	(35–86)	37	(31–46)	1.01	1.00	-	1.01	0.18
	ALT (U/L)^a^	11	(5–19)	9	(6–13)	1.03	1.00	-	1.07	0.09
	LD (U/L)^a^	524.5	(420–717)	421.5	(372–543)	1.00	1.00	-	1.00	0.03
	Glu (mg/dL)^a^	62.5	(41–78)	61.5	(50–75)	1.00	0.99	-	1.01	0.83
	CRP (mg/dL)^a^	5.185	(0.02–10.58)	0.012	(0.01–0.047)	2.07	1.35	-	3.17	<0.001
	Total IgM (mg/dL)^a^	114.5	(12–301)	8	(6–11)	1.07	1.01	-	1.13	0.02
	Total IgM > 20 (mg/dL)	30/40	75.0%	1/50	2.0%	147.00	17.91	-	1,206.74	<0.001

Abbreviations: OR, odds ratio; 95%CI, 95% confidence interval; IgM, immunoglobulin M; IM, intramuscular; IV, intravenous; PO, per Os; CRP, C-reactive protein; AST, aspartate aminotransferase; ALT, alanine aminotransferase; LD, lactate dehydrogenase.

a Median (interquartile range).

To avoid multicollinearity in the multivariate analysis, ‘failure to receive prenatal health checkups or irregular visits’, ‘unmarried at delivery’, and ‘maternal history as a commercial sex worker’ were excluded, as these three factors were strongly correlated with ‘maternal treatment not completed more than 4 weeks before delivery’, making it difficult to assess their independent effects. The authors also excluded some blood test results due to their non-specific nature. Based on these considerations, the following covariates were selected: maternal treatment not completed more than 4 weeks before delivery, total IgM > 20 mg/dL, preterm birth, and platelet count.

In the multivariable logistic regression analyses, maternal treatment not completed more than 4 weeks before delivery (OR: 7.20, 95% CI: 1.38–37.56, p = 0.02) and total IgM >20 mg/dL (OR: 65.31, 95% CI: 4.53–941.39, p = 0.002) were significantly associated with a higher risk of CS. (Table 5).

**Table 5.** A multivariate analysis of predictors of congenital syphilis

	OR	95%CI		*p*
Maternal treatment NOT completed more than 4 weeks before delivery	7.20	1.38	−37.56	0.02
Total IgM > 20 (mg/dL)	65.31	4.53	−941.39	0.002
Low birth weight	1.032	0.181	−5.892	0.97
Platelet count (10×4/μL)	0.94	0.87	−1.02	0.14

Abbreviations: OR, odds ratio; 95%CI, 95% confidence interval; IgM, immunoglobulin M.

### Comparison of serological findings as diagnostic indicators (Table 6)

The authors compared maternal and infant serological findings between cases with a definite or highly probable diagnosis and those with an excluded diagnosis. In CS cases, 93.8% (45/48) had a positive RPR, while only 25% (18/72) of the excluded cases had a positive RPR. Among the confirmed cases, 34.3% (12/35) and 11.4% (4/35) had an infant-to-mother RPR ratio of ≥1 and ≥ 4, respectively. In contrast, 97.0% (65/67) and 100% (67/67) of the excluded cases did not reach a ratio of ≥1 and ≥ 4, respectively, suggesting that high ratios are much less common in non-CS cases. The number of cases evaluated using infant RPR ≥1 was 48, whereas only 35 cases could be assessed using the infant-to-mother RPR ratio because of the limited availability of maternal RPR data.

**Table 6.** Comparison of serological findings

	Congenital syphilis			
	Definite (A) or highly probable (B) diagnosis		Excluded diagnosis (C)	
	n = 49	%	n = 73	%
Maternal RPR >4	36/43	83.7%	35/66	53.0%
Infant RPR≥1	45/48	93.8%	18/72	25.0%
Infant RPR/maternal RPR ≥1	12/35	34.3%	2/67	3.0%
Infant RPR/maternal RPR ≥4	4/35	11.4%	0/67	0.0%

Abbreviations: RPR, rapid plasma reagin.

## Discussion

This study is the first nationwide survey of pregnant women with syphilis and their newborns, including those with CS, conducted in Japan. Incomplete maternal treatment more than 4 weeks before delivery and infant total IgM > 20 mg/dL were found to be significant risk factors for suspected CS. Infant RPR titre, rather than the infant-to-mother RPR ratio, appears to be a more useful diagnostic indicator. Also, this study identified 41 cases between 2015 and 2024 that met the national surveillance of diagnosed CS definition for CS [25], accounting for 23.7% of the 173 cases reported during the same period, as documented in the national surveillance data [24]. The fact that the majority of pregnant women with syphilis in the present study had latent syphilis and were asymptomatic reinforces the importance of screening tests.

Of the two identified risk factors, ‘incomplete maternal treatment more than 4 weeks before delivery’ has already been acknowledged as a risk factor in prior literature [26]. It has been reported that in seropositive mothers with non-treponemal antibodies, if antimicrobial treatment is completed more than 4 weeks prior to delivery, most of their infants become seronegative within 6 months after birth, even without receiving treatment [27].

The second risk factor, ‘infant total IgM > 20 mg/dL’, is newly recognized. In this study, total IgM >20 mg/dL at birth showed an OR of 65 for CS. For early diagnosis of CS, FTA-ABS IgM can be measured, as it detects treponemal antibodies that do not cross the placenta. However, its insufficient sensitivity has been a concern [28–30]. Moreover, the assay is subjective, relies on visual interpretation by technicians, is not covered by the national health insurance system, and is no longer offered by many diagnostic laboratories. In contrast, total IgM can be measured promptly and objectively at many facilities using automated assays, though its positivity is not specific to syphilis and may reflect other congenital infections [31].

Among the examined potential risk factors, maternal history of sexually transmitted infections and marital status were not significantly associated with the outcome. In contrast, a history of sex industry employment and inadequate or no antenatal health checkups were significantly higher, with ORs of 5.33 and 3.91, respectively. Although these factors were excluded from multivariate analysis to avoid multicollinearity, they should still be considered important risk factors for CS. These two factors were closely related to and might be the underlying cause of maternal treatment not completed more than four weeks before delivery, which was also significantly associated with CS (Table 4). Prompt antimicrobial therapy and regular antenatal checkups were deemed most important in treating pregnant women with syphilis. Similar maternal risk factors, such as limited access to antenatal care and socio-economic vulnerability, have also been reported in studies from other high-income countries [32]. In addition, low birth weight has consistently been described as an adverse outcome associated with CS in these settings [33].

As a diagnostic indicator, the infant RPR titre was found to be more informative than the well-established infant-to-mother RPR ratio in this study [34–36]. Although the specificity was lower, the sensitivity was much higher. In addition, maternal information, including RPR data, is not always available, may be obtained from a different facility or test method, and the ratio cannot be calculated when the maternal RPR is negative. In fact, in this study, 30% of confirmed cases (14/49) had no available ratio data because the maternal RPR value was unknown. Of course, the traditional infant-to-mother RPR ratio may also be a useful indicator, as a significantly higher ratio may indicate a stronger likelihood of vertical transmission. Also, the ‘Proven or Highly Probable’ criterion has been defined as an infant RPR that is at least fourfold higher than the maternal RPR by the multiple dilution method, or 1.5 to 2.0 times higher by the automated method [12]. Typically, infant RPR titres are one to two times lower than maternal titres [34, 37]. This may be due to unknown reaction inhibitors in neonatal blood, limited placental transfer of maternal RPR IgM fractions (especially from early infection), or lower antibody levels in preterm infants due to incomplete placental transfer. However, since an elevated infant-to-mother RPR ratio inherently implies infant RPR positivity (≥1.0 R.U.), infant RPR positivity was considered more valuable for diagnosis.

This study has some limitations. First, as indicated by the finding that most infants with a negative RPR at birth were lost to follow-up, nearly half of the cases lacked sufficient diagnostic evaluation. Furthermore, it is recommended that infants with a negative infant RPR who do not receive treatment have their RPR retested at 3 months of age, and it is necessary to promote adherence to this recommendation by following a flowchart [12, 14]. Second, the risk factors were compared within the group of cases considered to have a potential for CS, rather than being compared with the general population of pregnant women. Third, if the mother contracts syphilis after her initial screening, syphilis-related antibody testing is not performed, potentially leading to missed cases of CS. However, universal syphilis screening for all neonates is not currently considered economically feasible. Fourth, more than half of the mothers were treated with amoxicillin with or without probenecid. This was because, while the international standard treatment is intramuscular benzathine penicillin G, it was not approved in Japan until 2022, necessitating the use of amoxicillin as an alternative. There is a report indicating that treatment with ampicillin or amoxicillin resulted in an overall CS occurrence rate of 14% and 33% in cases of late syphilis (including latent syphilis of unknown duration), respectively. However, no cases of CS were observed among mothers with early syphilis [38]. Nevertheless, with the recent introduction of intramuscular benzathine penicillin G, it is expected to become the standard treatment. Fifth, as this study was based on a survey of paediatricians, information on stillbirths and miscarriages was not available. However, although deceased individuals from infectious diseases are included in the criteria for notifiable disease surveillance in Japan [39], the number of reported deaths due to CS remains extremely low [40]. With the increasing incidence of syphilis in Japan, there is growing concern about a potential rise in the number of stillbirths, highlighting the need for high-precision surveillance.

## Conclusion

While uncertainties regarding maternal treatment during pregnancy remain, incorporating ‘maternal treatment not completed more than 4 weeks before delivery’ as a key criterion, together with the addition of total IgM levels, could facilitate the establishment of a more practical and effective treatment flow. Moreover, positive infant RPR, rather than the infant-to-mother RPR ratio, is likely to be more beneficial for evaluating CS.

## Supporting information

10.1017/S0950268825100629.sm001 Shimizu et al. supplementary material Shimizu et al. supplementary material

## Data Availability

The data presented are available upon reasonable request from the corresponding author after publication.

## Supplementary material

The supplementary material for this article can be found at http://doi.org/10.1017/S0950268825100629.

## References

[1] Trinh T, et al. (2019) Syphilis management in pregnancy: A review of guideline recommendations from countries around the world. Sexual and Reproductive Health Matters 27, 69–82. 31884900 10.1080/26410397.2019.1691897 PMC7888020

[2] Centers for Disease Control and Prevention (2022) *World Health Organization. Global Health Sector Strategies on, Respectively, HIV, Viral Hepatitis and Sexually Transmitted Infections for the Period 2022–2030.* Available at https://www.who.int/publications/i/item/9789240053779 (accessed 27 May 2025).

[3] Moseley P, et al. (2024) Resurgence of congenital syphilis: New strategies against an old foe. Lancet Infectious Diseases 24, e24–e35. 37604180 10.1016/S1473-3099(23)00314-6

[4] Takahashi T, et al. (2018) Rapid increase in reports of syphilis associated with men who have sex with women and women who have sex with men, Japan, 2012 to 2016. Sexually Transmitted Diseases 45, 139–143. 29420439 10.1097/OLQ.0000000000000768 PMC5815645

[5] National Institute of Infectious Diseases (2022) *National Epidemiological Surveillance of Infectious Diseases Annual Surveillance Data 2022.* Available at https://www.niid.go.jp/niid/ja/allarticles/surveillance/2270-idwr/nenpou/12558-syulist2022.html (accessed 27 May 2025).

[6] Kasamatsu A, et al. (2023) Unprecedented increase in syphilis cases among heterosexual men and women in Japan, 2021–2022. Sexual Health 20, 370–372. 37282345 10.1071/SH23031

[7] Ministry of Health, Labour and Welfare. *Demographic Survey.* Available at https://www.mhlw.go.jp/toukei/list/81-1a.html (accessed 27 May 2025).

[8] National Institute of Infectious Diseases (2024) *Syphilis in Pregnant Women and Congenital Syphilis in Japan, 2022–2023.* Available at https://www.niid.go.jp/niid/ja/syphilis-m-3/syphilis-idwrs/12628-syphilis-20240411.html (accessed 27 May 2025).

[9] Alexander JM, et al. (1999) Efficacy of treatment for syphilis in pregnancy. Obstetrics and Gynecology 93, 5–8. 9916946 10.1016/s0029-7844(98)00338-x

[10] Lin JS, Eder ML and Bean SI (2018) Screening for syphilis infection in pregnant women: Updated evidence report and systematic review for the US preventive services task force. JAMA 320, 918–925. 30193282 10.1001/jama.2018.7769 PMC13317666

[11] Fang J, et al. (2022) Congenital syphilis epidemiology, prevention, and management in the United States: A 2022 update. Cureus 14, e33009. 36712768 10.7759/cureus.33009 PMC9879571

[12] Ito Y, et al. (2025) Clinical practice guidelines for the management of Congenital Syphilis in Japan, 2023: Executive summary. Pediatric Infectious Disease Journal 44, e90–e94. 39714794 10.1097/INF.0000000000004665

[13] Arnold SR and Ford-Jones EL (2000) Congenital syphilis: A guide to diagnosis and management. Paediatrics & Child Health 5, 463–469. 20177559 10.1093/pch/5.8.463 PMC2819963

[14] Centers for Disease Control and Prevention (2021) *Congenital Syphilis.* Available at https://www.cdc.gov/std/treatment-guidelines/congenital-syphilis.htm (accessed 27 May 2025).

[15] Lee JH, et al. (2014) Comparison of an automated rapid plasma reagin (RPR) test with the conventional RPR card test in syphilis testing. BMJ Open 4, e005664. 10.1136/bmjopen-2014-005664 PMC4281540 25552608

[16] Tsuboi M, et al. (2018) Usefulness of automated latex Turbidimetric rapid plasma Reagin test for diagnosis and evaluation of treatment response in syphilis in comparison with manual card test: A prospective cohort study. Journal of Clinical Microbiology 56, e01003–e01018. 30135229 10.1128/JCM.01003-18 PMC6204675

[17] Shukla MR, et al. (2023) Evaluation of three automated Nontreponemal rapid plasma Reagin (RPR) tests for the laboratory diagnosis of syphilis. Journal of Clinical Microbiology 61, e0016823. 37219422 10.1128/jcm.00168-23 PMC10281131

[18] Arbefeville S, Lynch M and Ferrieri P (2019) Evaluation of a multiplex fully automated Treponemal and Nontreponemal (rapid plasma Reagin) assay. American Journal of Clinical Pathology 152, 230–236. 31139835 10.1093/ajcp/aqz034

[19] Official Journal of the European Union (2018) *Commission Implementing Decision (EU).* Available at https://eur-lex.europa.eu/legal-content/EN/TXT/PDF/?uri=CELEX:32018D0945&from=EN (accessed 27 May 2025).

[20] Herremans T, Kortbeek L and Notermans DW (2010) A review of diagnostic tests for congenital syphilis in newborns. European Journal of Clinical Microbiology and Infectious Diseases 29, 495–501. 20336337 10.1007/s10096-010-0900-8

[21] K DW (2021) Syphilis. Red Book: 2021–2024 Report of the Committee on Infectious Diseases. Itasca, IL: Amer Academy of Pediatrics, pp. 729–744

[22] Public Health Agency of Canada (2024) *National Case Definition: Congenital Syphilis.* Available at https://www.canada.ca/en/public-health/services/diseases/syphilis/health-professionals/national-case-definition-congenital-syphilis.html (accessed 27 May 2025).

[23] Workowski KA, et al. (2021) Sexually transmitted infections treatment guidelines, 2021. MMWR: Recommendations and Reports 70, 1–187. 10.15585/mmwr.rr7004a1 PMC8344968 34292926

[24] National Institute of Infectious Diseases (2024) *Summary of Syphilis Notifications in Japan.* Available at https://www.niid.go.jp/niid/images/epi/syphilis/2023q4/syphilis2023q4.pdf (accessed 27 May 2025).

[25] Infectious Agents Surveillance Report (2020) *Reporting Criteria for Syphilis.* Available at https://www.niid.go.jp/niid/images/iasr/36/420/de4201.pdf (accessed 27 May 2025).

[26] Thornton C, Chaisson LH and Bleasdale SC (2022) Characteristics of pregnant women with syphilis and factors associated with congenital syphilis at a Chicago hospital. Open Forum Infectious Diseases 9, ofac169. 35493123 10.1093/ofid/ofac169 PMC9045944

[27] Chang SN, et al. (1995) Seroreversion of the serological tests for syphilis in the newborns born to treated syphilitic mothers. Genitourinary Medicine 71, 68–70. 7744415 10.1136/sti.71.2.68 PMC1195455

[28] Scotti AT and Logan L (1968) A specific IgM antibody test in neonatal congenital syphilis. Journal of Pediatrics 73, 242–243. 4875771 10.1016/s0022-3476(68)80075-7

[29] Kaufman RE, Olansky DC and Wiesner PJ (1974) The FTA-ABS (IgM) test for neonatal congenital syphilis: A critical review. Journal of the American Venereal Disease Association 1, 79–84. 4616027

[30] Stoll BJ, et al. (1993) Clinical and serologic evaluation of neonates for congenital syphilis: A continuing diagnostic dilemma. Journal of Infectious Diseases 167, 1093–1099. 8486942 10.1093/infdis/167.5.1093

[31] Neu N, Duchon J and Zachariah P (2015) TORCH infections. Clinics in Perinatology 42, 77–103, viii. 25677998 10.1016/j.clp.2014.11.001

[32] Alberto C, et al. (2024) Syphilis in pregnant women and congenital syphilis from 2012 to 2021 in Switzerland: A multicentre, retrospective study. Swiss Medical Weekly 154, 3678. 39509430 10.57187/s.3678

[33] Carlson JM, et al. (2025) Birth outcomes among women with syphilis during pregnancy in six U.S. states, 2018–2021. Obstetrics and Gynecology 146, 121–128. 40536330 10.1097/AOG.0000000000005913 PMC13036627

[34] Rawstron SA and Bromberg K (1991) Comparison of maternal and newborn serologic tests for syphilis. American Journal of Diseases of Children 145, 1383–1388. 1669665 10.1001/archpedi.1991.02160120051018

[35] Palmeira P, et al. (2012) IgG placental transfer in healthy and pathological pregnancies. Clinical & Developmental Immunology 2012, 985646. 22235228 10.1155/2012/985646 PMC3251916

[36] Oloya S, et al. (2020) Prevalence, associated factors and clinical features of congenital syphilis among newborns in Mbarara hospital, Uganda. BMC Pregnancy and Childbirth 20, 385. 32616037 10.1186/s12884-020-03047-y PMC7330944

[37] Stoll BJ (1994) Congenital syphilis: Evaluation and management of neonates born to mothers with reactive serologic tests for syphilis. Pediatric Infectious Disease Journal 13, 845–852; quiz 853. 7854881

[38] Nishijima T, et al. (2020) Effectiveness and tolerability of oral amoxicillin in pregnant women with active syphilis, Japan, 2010–2018. Emerging Infectious Diseases 26, 1192–1200. 32441638 10.3201/eid2606.191300 PMC7258477

[39] Ministry of Health, Labour and Welfare. *Notification of Physicians and Veterinarians under the Infectious Disease Control Law.* Available at https://www.mhlw.go.jp/bunya/kenkou/kekkaku-kansenshou11/01-05-11.html (accessed 3 June 2025).

[40] Japan Association of Obstetricians and Gynecologist (2024) Report on Survey Results Regarding Syphilis during Pregnancy *(*2023*).* Available at https://www.jaog.or.jp/wp/wp-content/uploads/2024/01/671a5569ce325c0b02e8b1e65d5eaa03.pdf (accessed 3 June 2025).

