# Supplementary material for: Risk factors and diagnostic indicators for congenital syphilis: a Nationwide retrospective survey
Source: Epidemiol Infect. 2025 Oct 22;153:e127. doi: 10.1017/S0950268825100629 (PMC12641297; doi:10.1017/S0950268825100629)
Supplement: Supplementary file 1 [file S0950268825100629sup001.docx]

Department of Pediatrics	Okinawa Chubu Hospital	Okinawa
Department of Pediatric Emergency Medicine	Osaka City General Hospital	Osaka
Department of Perinatal and Pediatric Infectious Diseases	Osaka Women's and Children's Hospital	Osaka
Division of Neonatology, Department of Pediatrics	Saitama City Hospital	Saitama
Department of Infectious Diseases and Immunology, Allergy	Saitama prefectural Children's Medical Center	Saitama
Department of Pediatrics	Sapporo Medical University Hospital	Hokkaido
Department of Pediatrics	National Hospital Organization Shikoku Medical Center for Children and Adults	Kagawa
Division of Infectious Diseases, Department of Pediatrics	Tokyo Metropolitan Children's Medical Center	Tokyo
Department of Neonatology	Tokyo Womens' Medical University Hospital	Tokyo
Department of Pediatrics	University of Occupational and Environmental Health	Fukuoka
Children's Medical Center	Yokohama City University Medical Center	Kanagawa
Department of Pediatrics	Yokohama Municipal Citizen's Hpspital	Kanagawa
